# Numerical Interchain Mean-Field Theory for the Specific Heat of the Bimetallic Ferromagnetically Coupled Chain Compound MnNi(NO_2_)_4_(en)_2_ (en = Ethylenediamine)

**DOI:** 10.3390/molecules27196546

**Published:** 2022-10-03

**Authors:** Andreas Honecker, Wolfram Brenig, Maheshwor Tiwari, Ralf Feyerherm, Matthias Bleckmann, Stefan Süllow

**Affiliations:** 1 Laboratoire de Physique Théorique et Modélisation, CNRS UMR 8089, CY Cergy Paris Université, 95302 Cergy-Pontoise, France; 2 Institut für Theoretische Physik, TU Braunschweig, 38106 Braunschweig, Germany; 3 Helmholtz-Zentrum Berlin für Materialien und Energie GmbH, 14109 Berlin, Germany; 4 Institut für Physik der Kondensierten Materie, TU Braunschweig, 38106 Braunschweig, Germany; 5 Wehrwissenschaftliches Institut für Werk- und Betriebsstoffe (WIWeB), 85435 Erding, Germany

**Keywords:** quantum spin chains, specific heat, quantum Monte Carlo simulations, exact diagonalization, mean-field theory

## Abstract

We present a detailed study of the field-dependent specific heat of the bimetallic ferromagnetically coupled chain compound MnNi(NO${}_{2}$)${}_{4}$(en)${}_{2}$, en = ethylenediamine. For this material, which in zero field orders antiferromagnetically below $T_{N}=2.45$ K, small fields suppress magnetic order. Instead, in such fields, a double-peak-like structure in the temperature dependence of the specific heat is observed. We attribute this behavior to the existence of an acoustic and an optical mode in the spin-wave dispersion as a result of the existence of two different spins per unit cell. We compare our experimental data to numerical results for the specific heat obtained by exact diagonalization and Quantum Monte Carlo simulations for the alternating spin-chain model, using parameters that have been derived from the high-temperature behavior of the magnetic susceptibility. The interchain coupling is included in the numerical treatment at the mean-field level. We observe remarkable agreement between experiment and theory, including the ordering transition, using previously determined parameters. Furthermore, the observed strong effect of an applied magnetic field on the ordered state of MnNi(NO${}_{2}$)${}_{4}$(en)${}_{2}$ promises interesting magnetocaloric properties.

## 1. Introduction

Alternation in spin systems, be it of the magnetic coupling, the local symmetry, or the spin value, induces new and exotic types of magnetic ground states and excitations [1,2,3,4,5,6,7,8,9,10,11,12,13,14,15,16,17,18]. In particular, this is exemplified in novel bimetallic chain systems, *viz.* molecule-based chain systems with alternately arranged magnetic units carrying quantum spins $S_{1}$ and $S_{2}$ of different sizes. The ability to synthesize mixed-spin-chain materials [9,19,20,21,22,23,24,25,26,27] has stimulated theoretical investigations [10,11,12,13,14,15,16,17,18,28,29,30,31,32,33,34,35]. The magnon dispersion relation of such chains splits into an optical and an acoustic mode because of the two differently sized quantum spins $S_{1}$ and $S_{2}$ per unit cell, both for antiferromagnetic coupling along the chain [10,11,12,13,14,15,16,17,35] as well as ferromagnetic coupling [18,28,29,32,33,34]. Although ground state and fundamental excitations of a Heisenberg ferromagnet are simple, thermodynamic properties are very sensitive to interactions of the magnon excitations, as is evidenced by the ferromagnetic uniform spin-1/2 Heisenberg chain (compare chapter 11.3 of [36] and references therein). Computations for bimetallic Heisenberg chains show that the two energy scales associated to the acoustic and the optical spin excitation modes are reflected by “double-peak” kind of features in the specific heat $c_{p}\left(T\right)$ for both antiferro- [10,11,12,13,14,15,16,17] and ferromagnetic [18,28,29] coupling.

Similar predictions have been made for chains of mixed classical and quantum spins as far back as 1975 [37]. In spite of this long history, experimental verification of the features expected in the specific heat is lacking. In fact, experimental tests of mixed-spin-chain models are scarce [38,39,40], since most materials available contain elements with larger spins [20,21,22,41,42,43,44], which are difficult to be treated adequately in theoretical calculations [10,11,12,13,14,15,16,17].

Here, we present a verification of the two energy scale prediction via a detailed study of the specific heat $c_{p}$ of MnNi(NO${}_{2}$)${}_{4}$(en)${}_{2}$, en = ethylenediamine = C${}_{2}$N${}_{2}$H${}_{4}$, in zero and applied fields. After the field-induced suppression of long-range antiferromagnetic order, we observe a double-peak-like structure in the temperature dependence of $c_{p}$ for MnNi(NO${}_{2}$)${}_{4}$(en)${}_{2}$. We compare our findings with the results of numerical calculations for an $S_{1}=1$, $S_{2}=5/2$ mixed spin chain in zero and external fields. We demonstrate that the in-field calculations, for which finite-size effects are negligible, fully reproduce the double-peak structure of the experimentally observed in-field specific heat. This shows that the optical and acoustic spin excitation mode are reflected by the thermodynamics of this bimetallic chain system. Quantum Monte Carlo (QMC) simulations of the individual chains augmented by a self-consistent mean-field treatment of interchain coupling even yields a remarkably accurate description of the ordering transition in a vanishing magnetic field.

The remainder of this manuscript is organized as follows: Section 2 presents more details on MnNi(NO${}_{2}$)${}_{4}$(en)${}_{2}$ and, in particular, a measurement of its specific heat. We then proceed in Section 3 with a detailed theoretical analysis based on exact diagonalization and QMC simulations combined with a mean-field treatment of interchain coupling; some complementary details are provided in Appendix B. In Section 4, we briefly comment on the magnetocaloric properties of MnNi(NO${}_{2}$)${}_{4}$(en)${}_{2}$ before we summarize our findings in Section 5. Appendix C contains a summary of a complementary single-site mean-field treatment.

## 2. Experiment

### 2.1. MnNi(NO${}_{2}$)${}_{4}$(en)${}_{2}$ (en = Ethylenediamine)

**Figure 1:**
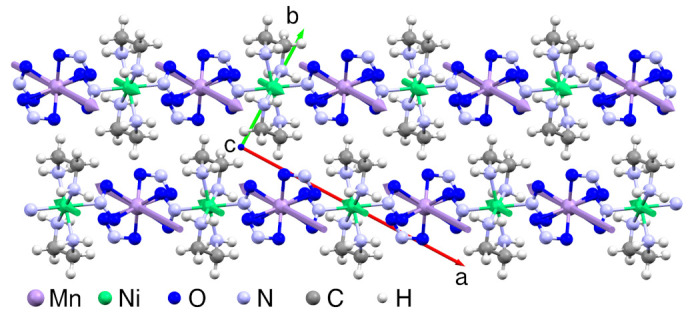
Two chains in the a-b plane of MnNi(NO${}_{2}$)${}_{4}$(en)${}_{2}$, based on the crystal structure of [45]. The thick arrows on the Mn and Ni atoms show the zero-field ordered state: ferromagnetic along the chains and antiferromagnetic between chains.

MnNi(NO${}_{2}$)${}_{4}$(en)${}_{2}$ is one of the best characterized mixed spin chain compounds [45,46,47,48], crystallizing in an orthorhombic structure, space group *Pccn* (lattice parameters a=14.675 Å, b=7.774 Å, c=12.401 Å). It contains chains of alternately arranged Ni and Mn ions linked by NO${}_{2}$ ligands, which carry magnetic moments with spin $S_{1}=1$ and $S_{2}=5/2$, respectively (Figure 1). The magnetic coupling along the chain, *J*, is ferromagnetic [47], with J=2.8 K [28] (we will use units such that $k_{B}=1$ throughout.). A finite ionic zero-field splitting *D* of 0.36 K is derived from the anisotropy of the susceptibility. Due to an effective antiferromagnetic interchain coupling of $J_{⊥}=0.036$ K, the system undergoes a transition into an antiferromagnetically (AFM) ordered state below $T_{N}=2.45$ K in zero magnetic field and at ambient pressure [28,47,48]. The long-range magnetically ordered state is suppressed by rather small magnetic fields [47].

### 2.2. Specific Heat

For our study, we used single crystals of MnNi(NO${}_{2}$)${}_{4}$(en)${}_{2}$ investigated previously [47] that were grown by slow evaporation, as described in detail in Ref. [46]. Here, we present the easy-axis data B∥c, for which AFM ordering is suppressed in ∼0.3 T. The heat capacity was measured using commercial calorimeters in magnetic fields B∥c up to 1.6 T at temperatures *T* down to 0.4 K. As will be discussed below, these *c* axis data allow a comparison to more accurate numerical calculations than the data ∥a.

**Figure 2:**
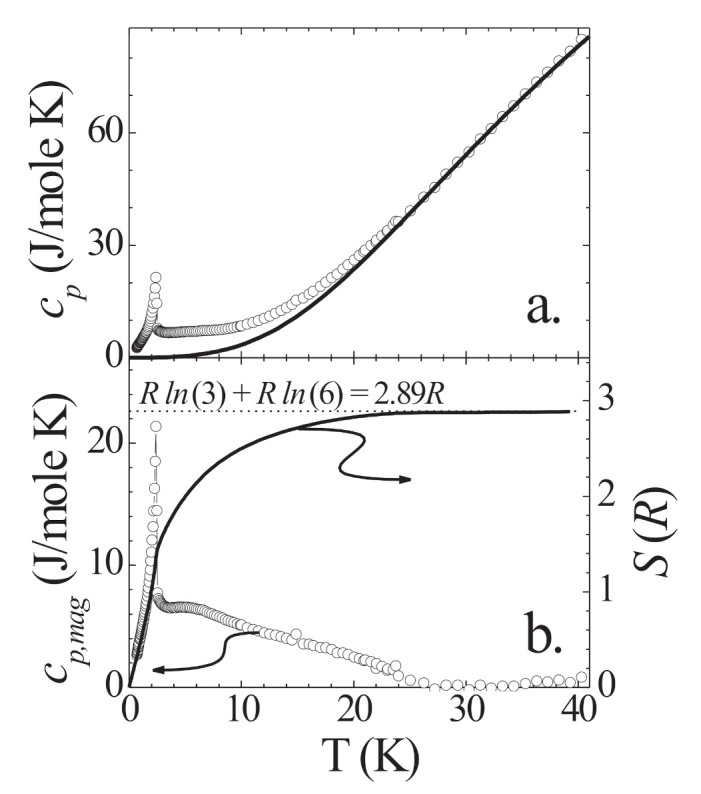
(**a**) Zero-field specific heat $c_{p}$ of MnNi(NO${}_{2}$)${}_{4}$(en)${}_{2}$ as function of temperature *T*. (**b**) Zero-field magnetic specific heat $c_{p,mag}$ and associated entropy *S* per mole of MnNi(NO${}_{2}$)${}_{4}$(en)${}_{2}$ as function of temperature.

In Figure 2a, we depict the zero-field specific heat $c_{p}$ of MnNi(NO${}_{2}$)${}_{4}$(en)${}_{2}$ as a function of *T*. The AFM anomaly at $T_{N}=2.45$ K is clearly discernible. To derive the magnetic specific heat, we determine the lattice contribution $c_{p,lat}$. Since a single $T^{3}$-term does not reproduce the experimental data above $T_{N}$, we use two Debye contributions, each calculated via the full Debye integral, to parametrize $c_{p,lat}$. MnNi(NO${}_{2}$)${}_{4}$(en)${}_{2}$ is built up by chain segments -Mn-NO${}_{2}$-Ni-NO${}_{2}$-, with two ethylenediamine molecules and two NO${}_{2}$ groups attached to the Mn and Ni ions, respectively (Figure 1). Intramolecular oscillations of ethylenediamine or NO${}_{2}$, because of the light atoms involved, yield Einstein contributions, which are irrelevant for the temperatures considered here. The chain segment units Mn, Ni, and NO${}_{2}$ are similar in atomic weight. Therefore, to parametrize the lattice contribution of these units we choose one Debye temperature $\Theta_{D}$ with 3×4=12 modes. Analogously, the four attached molecules ethylenediamine and NO${}_{2}$ per chain segment are parametrized by a second Debye temperature contributing with 12 modes. This way, we reproduce the lattice specific heat of MnNi(NO${}_{2}$)${}_{4}$(en)${}_{2}$ with Debye temperatures $\Theta_{D1}=138$ K and $\Theta_{D2}=249$ K (solid line in Figure 2a).

We obtain the magnetic specific heat contribution $c_{p,mag}$ by subtracting $c_{p,lat}$ from the total $c_{p}$ (Figure 2b). Further, by numerically integrating $c_{p,mag}/T$, we obtain the magnetic entropy *S* included in Figure 2b. Both quantities indicate that above $T_{N}$ there are magnetic fluctuations present over a wide temperature range. In $c_{p,mag}$, there is a broad anomaly ranging up to $∼10\;T_{N}$. The associated entropy reaches only 1.4R at $T_{N}$, which is less than half of the value expected for the sum of the magnetic entropies of Ni ($S_{1}=1$) and Mn ($S_{2}=5/2$), Rln(3)+Rln(6)≈2.89R (dotted line in Figure 2b). This value is reached only at 10 $T_{N}$. Note that the saturation of *S* at 2.89R demonstrates the consistency and adequacy of our derivation of the lattice specific heat.

**Figure 3:**
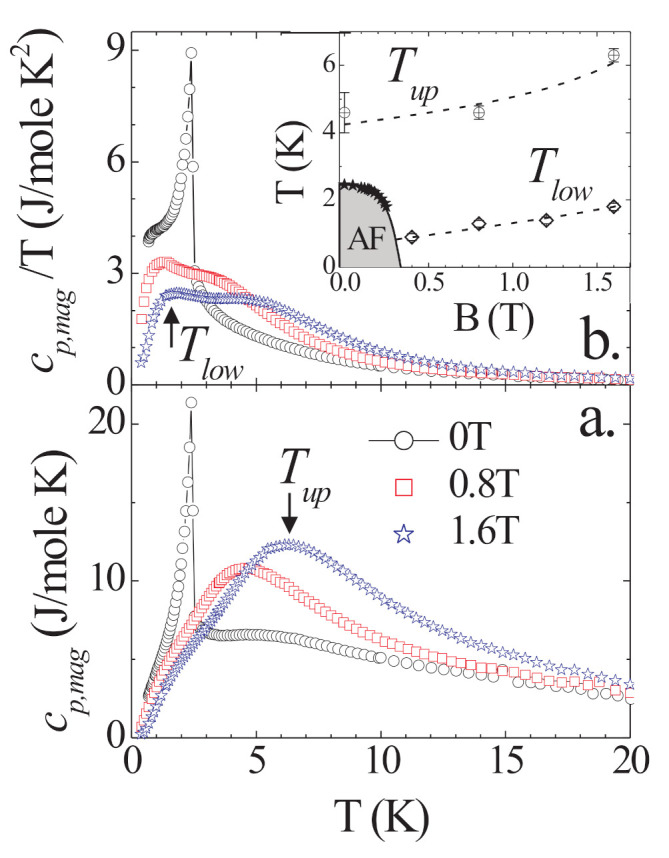
(**a**) Field dependence of $c_{p,mag}$ of MnNi(NO${}_{2}$)${}_{4}$(en)${}_{2}$ for fields B∥c. (**b**) The same data plotted as $c_{p,mag}/T$. Inset: The magnetic phase diagram of MnNi(NO${}_{2}$)${}_{4}$(en)${}_{2}$ for B∥c: $T_{N}$ from Ref. [47] (🟉), $T_{\mathrm{up}}$ from the maximum in $c_{p,mag}$ (⊕), $T_{\mathrm{low}}$ from the maximum in $c_{p,mag}/T$ (⋄); lines are guides to the eye.

AFM order in MnNi(NO${}_{2}$)${}_{4}$(en)${}_{2}$ is suppressed by small magnetic fields [47]. This enables us to study magnetic fluctuations in MnNi(NO${}_{2}$)${}_{4}$(en)${}_{2}$, as they appear in $c_{p}$. In Figure 3, we plot $c_{p,mag}$ as function of field. We observe a rapid suppression of the AFM state, in agreement with Ref. [47]. Moreover, after suppression of the AFM state, the broad specific heat anomaly above $T_{N}$ becomes much more pronounced in magnetic fields and is clearly visible already in the non-phonon corrected data.

The temperature $T_{\mathrm{up}}$ of the maximum in $c_{p,mag}$ represents a measure for an energy scale characteristic for the magnetic fluctuation spectrum (indicated for the 1.6 T data in Figure 3). In the inset of Figure 3, we record its field dependence up to 1.6 T, with a modest increase in $T_{\mathrm{up}}$ of about 1 K/T. Further, after suppression of AFM order in the *T* dependence of $c_{p}$ there is additional structure. This is most clearly seen for $c_{p,mag}/T$, where one now observes a double-peak-like structure (see Figure 3b). We take as measure for a second characteristic energy scale $T_{\mathrm{low}}$ the maximum in $c_{p,mag}/T$ and include its field dependence in Figure 3. Again, we find a modest increase of $T_{\mathrm{low}}$ by about 1 K between 0.4 and 1.6 T.

$T_{\mathrm{up}}$ and $T_{\mathrm{low}}$ are clearly distinct temperatures and increase at a similar rate. Therefore, they do not stem from ionic states Zeeman split in an external field. Further, extrapolating $T_{\mathrm{low}}$ to zero field yields a finite value of about 0.7 K, implying that $T_{\mathrm{low}}$ does not arise from Zeeman splitting of ionic degenerate states. Therefore, we associate both characteristic energy scales $T_{\mathrm{up}}$ and $T_{\mathrm{low}}$ with collective excitation modes of the magnetic fluctuation spectrum of MnNi(NO${}_{2}$)${}_{4}$(en)${}_{2}$ as the result of the existence of an acoustic and an optical magnon mode.

## 3. Theory

We now proceed to provide a theoretical description of the experimental findings.

### 3.1. Model

We start from the basic chain model

(1) $$H=-J\sum_{x=1}^{N/2}\left({\vec{S}}_{x}\cdot {\vec{s}}_{x}+{\vec{s}}_{x}\cdot {\vec{S}}_{x+1}\right)-D\sum_{x=1}^{N/2}{\left(S_{x}^{z}\right)}^{2}-h\sum_{x=1}^{N/2}\left(S_{x}^{z}+s_{x}^{z}\right)\;,$$

where the ${\vec{s}}_{x}$ (${\vec{S}}_{x}$) correspond to the spins of the Ni ions (Mn ions) and have $S_{1}=1$ ($S_{2}=5/2$). Following Refs. [28,47], we take a single-ion anisotropy into account only for the Mn sites. The main role of this anisotropy is to select a preferred axis, it should not matter too much if this is due to the Mn or the Ni sites, and it is the form Equation (1) for which parameters were extracted in Ref. [28] by analyzing the high-temperature behavior of the magnetic susceptibility. Nevertheless, we refer to Appendix A for a discussion of the one-magnon dispersion for the case where both anisotropies are present. In the following discussion, we will use the parameters that have been determined in Ref. [28]—namely, J=2.8 K and D=0.36 K—or in units with J=1: D=0.36/2.8≈0.129. In the latter units, and assuming magnetic *g* factors g=2, the magnetic fields of 0.8 T and 1.6 T shown in Figure 3 are modeled by h=0.4 and 0.8, respectively.

### 3.2. Numerical Treatment of Decoupled Chains

Previously, some of the present authors have performed exact (full) diagonalization and Quantum Monte Carlo (QMC) simulations of chains with $S_{1}=1/2$, $S_{2}=1$ [28,29]. The previous exact diagonalization (ED) investigations went to N=14 spins with $S_{1}=1/2$ and $S_{2}=1$. When we replace a spin 1/2 by 5/2, the local Hilbert space dimension increases from 2 to 6, i.e., by a factor 3. Thus, here, we have to contend ourselves with ED for chains with N=10. Adding one unit cell would increase the total Hilbert space dimension by a factor of 18 for the case $S_{1}=1$, $S_{2}=5/2$ such that the next system size N=12 remains out of reach. We use conservation of $S^{z}$ as well as spatial symmetries. The magnetic susceptibility χ and specific heat *c* can then be calculated from the eigenvalues and the associated quantum numbers.

To access longer chains, we use QMC. The present QMC simulations were carried out with the ALPS [49,50] directed loop applications [51,52] in the stochastic series expansion framework [53]. To be precise, these computations were started a while ago. Therefore, we used version 1.3 of the ALPS applications [50] rather than the more recent release 2.0 [54]. The specific heat in a magnetic field can be sensitive to the pseudorandom number generator; so, this needs to be carefully chosen. Here, we used the “Mersenne Twister 19937” pseudorandom number generator [55]. To verify reliability of our results, we performed QMC simulations for N=10 (data not shown here) and double-checked them against our ED computations for the same system size.

**Figure 4:**
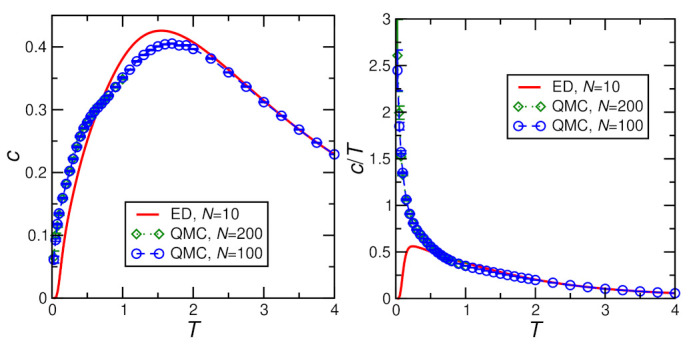
Specific heat per spin *c* calculated for D=0, h=0, J=1 by exact diagonalization (ED) and Quantum Monte Carlo (QMC). The left panel shows the specific heat itself while the right panel shows the specific heat divided by temperature c/T.

**Figure 5:**
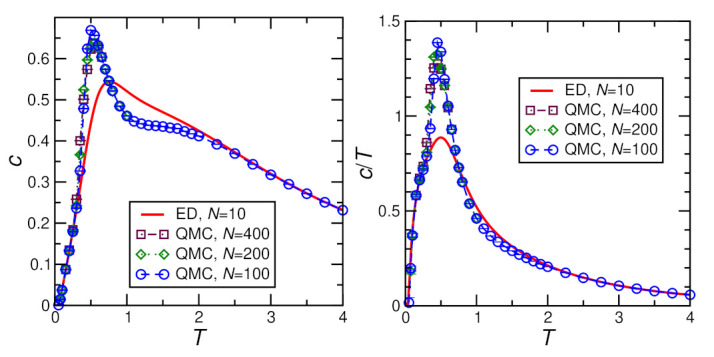
Specific heat per spin *c* calculated for D=0.36/2.8, h=0, J=1. As in Figure 4, the left panel shows the specific heat itself while the right panel shows the specific heat divided by temperature c/T.

**Figure 6:**
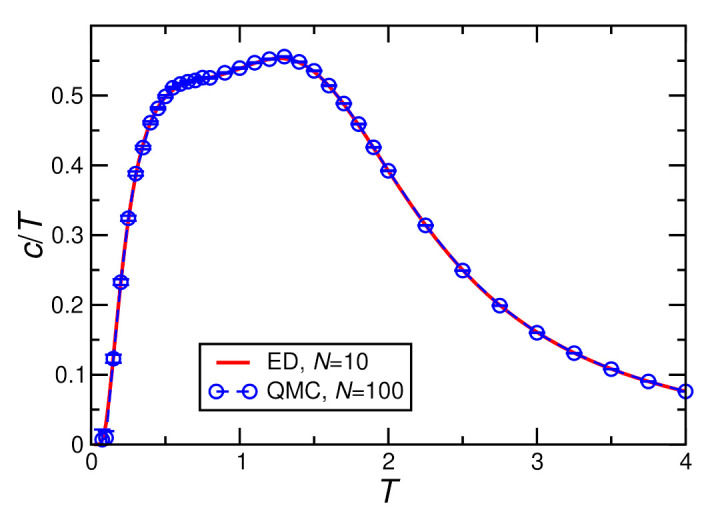
Specific heat per spin divided by temperature c/T calculated for D=0.36/2.8, J=1 in a magnetic field h=0.4.

**Figure 7:**
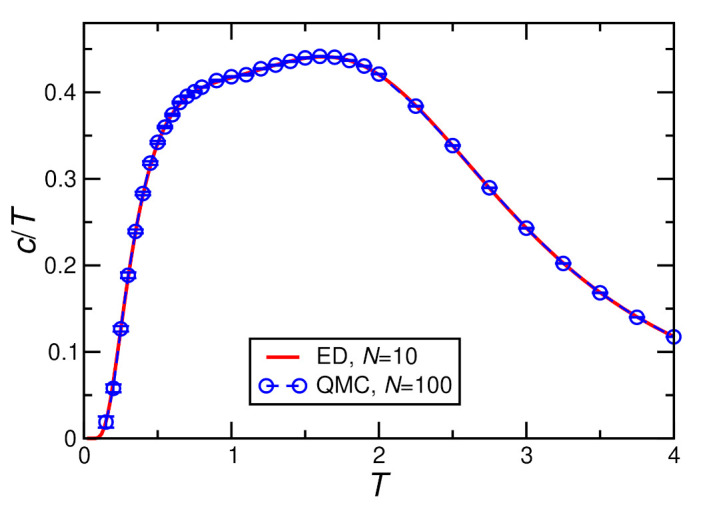
Specific heat per spin divided by temperature c/T calculated for D=0.36/2.8, J=1 in a magnetic field h=0.8.

Figure 4, Figure 5, Figure 6 and Figure 7 show ED (N=10) and QMC (N≥100) results for the specific heat. The QMC simulations become challenging at the lowest temperatures, in particular for finite *D* and *h*. This leads to visible statistical error bars at low *T*, in particular, in Figure 6 and Figure 7; otherwise, statistical errors are negligible. For h=0, finite-size effects are relevant, as demonstrated by visible deviations between the N=10 and 100 data in Figure 4 and Figure 5. On the other hand, no further change is visible for larger *N*, i.e., N=100 can be considered as representative of the thermodynamic limit for h=0. Finally, a field of h≥0.4J lifts the ground-state degeneracy and opens a sufficiently large gap in the spectrum such that N=10 and N=100 become indistinguishable (see Figure 6 and Figure 7) and N=10 ED suffices to describe the thermodynamic limit.

For h=0 and D=0, the ground state is an SU(2) multiplet with (7N/2+1) components. This leads to a difference between the zero-temperature entropies per site for N=10 and N=100 of ΔS=0.299744… Accordingly, the entropy integral $\int_{0}^{\infty }dT\;c/T$, i.e., the corresponding area under the N=100 curve of the right panel of Figure 4, is expected to be bigger than that of the corresponding N=10 curve by this amount ΔS. The QMC data for the specific heat *c* not only exhibit a maximum at T≈1.8J but also a shoulder at T≈0.5J (see left panel of Figure 4), corresponding to the two expected features [28,29].

Figure 5 shows the result with the single-ion anisotropy D>0 included, still at h=0. The presence of the single-ion anisotropy reduces the ground-state degeneracy to two and opens a gap in the one-magnon spectrum, see Appendix A for details. For N=10, the resulting ground-state entropy ln2 is still almost 5% of the total entropy. This leads to a difference between the zero-temperature entropies per site for N=10 and N=100 of ΔS=ln2/10−ln2/100=0.062383… While this is smaller than in the case of D=0, the difference is still visible in the ED data compared with those of QMC, shown in the right panel of Figure 5. From the point of view of physics, the specific heat *c* in the left panel of Figure 5 may be more instructive. The shoulder-like feature for D=0 developed into a sharp peak around T≈0.5J for the value D=0.36/2.8 while, in turn, the previous global maximum of *c* became a shoulder around T≈1.7J. In any case, these two features can be traced from D=0 to finite *D*.

Finally, we add a magnetic field h>0, corresponding to the experimental case where we actually observed two features in the specific heat (see Figure 3b). Application of a finite field h>0 not only lifts the remaining ground-state degeneracy, but h≥0.4J opens a sufficiently large gap in the spectrum such that finite-size effects are negligible already for N=10, as mentioned before and shown in Figure 6 and Figure 7. As in the experiment, we observe the emergence of a double-peak structure where both the feature at T≈0.5J and in particular the one at T/J=1.5…2 shift to higher temperatures with increasing magnetic field (compare Figure 6 and Figure 7).

### 3.3. Mean-Field Treatment of the Interchain Coupling

In zero external magnetic field, an antiferromagnetic phase transition with a Néel temperature $T_{N}=2.45\;K=0.875\;J$ is observed experimentally, as discussed in Section 2. This demonstrates that interchain coupling should be included in a quantitative description, at least for h=0 and T≲J, even if the numerical results of Section 3.2 already qualitatively reproduce the experiment in a finite magnetic field.

Since the chains are ferromagnetic, we assume that only the total magnetization of one chain acts via an effective field on the neighboring chains. The assumption of only average magnetizations of one chain affecting the neighboring ones is motivated by the exact exchange paths between chains in MnNi(NO${}_{2}$)${}_{4}$(en)${}_{2}$ being unknown (compare the crystal structure of Figure 1) and was also made in Ref. [28]. To be precise, one starts from a coupling between chains *i* and *j* of the form

(2) $$\frac{J_{i,j}}{2\;N}\sum_{x=1}^{N/2}\left({\vec{S}}_{i,x}+{\vec{s}}_{i,x}\right)\cdot \sum_{y=1}^{N/2}\left({\vec{S}}_{j,y}+{\vec{s}}_{j,y}\right)$$

which one replaces by

(3) $$\frac{J_{i,j}}{2}\;\langle M_{i}\rangle \sum_{y=1}^{N/2}\left(S_{j,y}^{z}+s_{j,y}^{z}\right)+\frac{J_{i,j}}{2}\;\langle M_{j}\rangle \sum_{x=1}^{N/2}\left(S_{i,x}^{z}+s_{i,x}^{z}\right)-N\;\frac{J_{i,j}}{2}\;\langle M_{i}\rangle \;\langle M_{j}\rangle \;.$$

We drop the term $-N\;\frac{J_{i,j}}{2}\;\langle M_{i}\rangle \;\langle M_{j}\rangle$ for the time being, but one should remember to add this term for total energy computations and in particular if one wants to write expectation values as derivatives of the free energy, see also Ref. [56]. This leads to a family of interchain mean-field Hamiltonians

(4) $$H_{i}^{\mathrm{MF}}=-J\sum_{x=1}^{N/2}\left({\vec{S}}_{x}\cdot {\vec{s}}_{x}+{\vec{s}}_{x}\cdot {\vec{S}}_{x+1}\right)-D\sum_{x=1}^{N/2}{\left(S_{x}^{z}\right)}^{2}-\left(h-\sum_{j\neq i}J_{i,j}\;\langle M_{j}\rangle \right)\sum_{x=1}^{N/2}\left(S_{x}^{z}+s_{x}^{z}\right)\;,$$

where the magnetization of the *i*th chain should satisfy the self-consistency condition

(5) $$N\;\langle M_{i}\rangle =\frac{\mathrm{Tr}\left(\sum_{x=1}^{N/2}\left(S_{x}^{z}+s_{x}^{z}\right)\;e^{-\beta \;H_{i}^{\mathrm{MF}}}\right)}{\mathrm{Tr}\left(e^{-\beta \;H_{i}^{\mathrm{MF}}}\right)}=\frac{\mathrm{Tr}\left(\sum_{x=1}^{N/2}\left(S_{x}^{z}+s_{x}^{z}\right)\;e^{-\beta \;\left(H_{i}^{\mathrm{MF}}-N\;\sum_{j\neq i}\frac{J_{i,j}}{2}\;\langle M_{i}\rangle \;\langle M_{j}\rangle \right)}\right)}{\mathrm{Tr}\left(e^{-\beta \;\left(H_{i}^{\mathrm{MF}}-N\;\sum_{j\neq i}\frac{J_{i,j}}{2}\;\langle M_{i}\rangle \;\langle M_{j}\rangle \right)}\right)}$$

with β=1/T (recall that we chose units such that $k_{B}=1$).

We now consider two cases. Firstly, for h=0, we expect antiferromagnetic order that should be described by two types of chains i=1,2. Furthermore, by symmetry, one expects that $\langle M_{1}\rangle =-\langle M_{2}\rangle =\langle M\rangle$. This sign difference can be absorbed by a spin inversion on every other chain, which also flips the sign of the interchain coupling. Therefore, we introduce an effective interchain coupling $J_{⊥}=-\sum_{j\neq i}J_{i,j}$, where the minus sign will allow us to treat all chains as having the same magnetization 〈M〉≥0. Secondly, for h≥0.4, one stays in a paramagnetic phase where we expect all chain magnetizations to be equal $\langle M_{i}\rangle =\langle M\rangle$. Now, we straightforwardly set the effective interchain coupling $J_{⊥}=\sum_{j\neq i}J_{i,j}$.

Under either of these assumptions, the family of mean-field Hamiltonians (4) reduces to a single interchain mean-field Hamiltonian

(6) $$H^{\mathrm{MF}}=H_{1D}-\left(h-J_{⊥}\;\langle M\rangle \right)\;N\;M\;,$$

with

(7) $$H_{1D}=-J\sum_{x=1}^{N/2}\left({\vec{S}}_{x}\cdot {\vec{s}}_{x}+{\vec{s}}_{x}\cdot {\vec{S}}_{x+1}\right)-D\sum_{x=1}^{N/2}{\left(S_{x}^{z}\right)}^{2}\;,\;N\;M=\sum_{x=1}^{N/2}\left(S_{x}^{z}+s_{x}^{z}\right)\;.$$

The magnetization should now satisfy the modified self-consistency condition

(8) $$\langle M\rangle =\frac{\mathrm{Tr}\left(M\;e^{-\beta \;H^{\mathrm{MF}}}\right)}{\mathrm{Tr}\left(e^{-\beta \;H^{\mathrm{MF}}}\right)}=\frac{\mathrm{Tr}\left(M\;e^{-\beta \;\left(H^{\mathrm{MF}}-N\;\frac{J_{⊥}}{2}{\langle M\rangle }^{2}\right)}\right)}{\mathrm{Tr}\left(e^{-\beta \;\left(H^{\mathrm{MF}}-N\;\frac{J_{⊥}}{2}{\langle M\rangle }^{2}\right)}\right)}\;.$$

Recall that in order to cast both the antiferromagnetic case at h=0 and the paramagnetic case at h>0 in the same single-chain form, it was necessary to introduce different signs for the effective interchain coupling $J_{⊥}$ in the two cases. Still, the absolute value of $J_{⊥}$ is the same in both cases.

**Figure 8:**
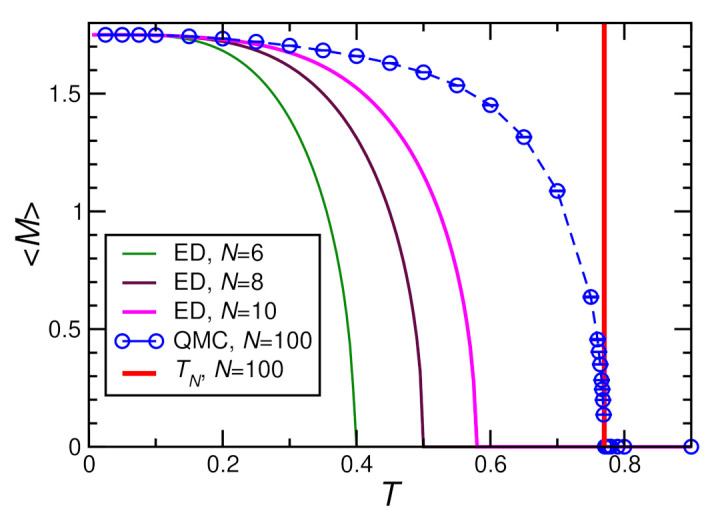
Self-consistent mean-field results for the magnetization 〈M〉 with $J_{⊥}=-0.072/2.8$, D=0.36/2.8, h=0, J=1. The vertical line indicates the estimate $T_{N}=0.77$ for the Néel temperature obtained from chains with N=100 sites (for details, see text).

**Figure 9:**
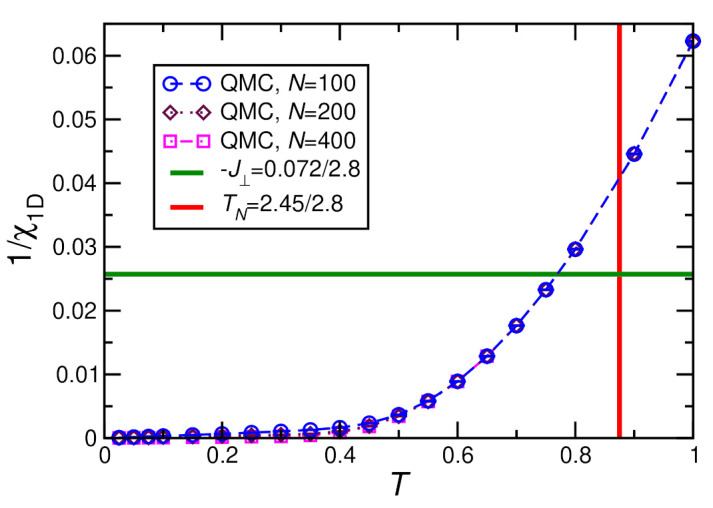
Inverse magnetic susceptibility calculated by QMC for a single chain with D=0.36/2.8, h=0, J=1. Furthermore, also shown are the estimated value of the interchain coupling $-J_{⊥}\;=\;0.072$ K [28] as well as the experimental Néel temperature $T_{N}=2.45$ K [28,47] divided by J=2.8 K.

Since the magnetization 〈M〉 is easily evaluated even within QMC, it is possible to run a self-consistency loop using a numerical evaluation of the chain magnetization 〈M〉, i.e., one starts with an initial guess such as 〈M〉=7/4, recomputes 〈M〉 from Equation (8), and iterates until a desired level of accuracy is reached (compare Appendix B for further details). Some ED and QMC results for the self-consistent 〈M〉 for h=0 are shown in Figure 8. The vertical line in Figure 8 shows an estimate of the Néel temperature that will be discussed in the following Section 3.3.1. One observes in Figure 8 that the estimated Néel temperature $T_{N}$ varies by almost a factor two as one goes from N=6 to 100 spins in a chain. Even with N=10, one still deviates by about 25% from the estimate obtained with N=100. On the other hand, analysis of the data shown in Figure 5 and Figure 9 below indicates that N=100 should indeed be sufficient to represent the thermodynamic limit along the chains.

#### 3.3.1. Magnetic Susceptibility and Ordering Temperature

The numerical treatment of a single chain yields direct access to

(9) $$\chi_{1D}=\beta \;N\;\left(\langle M^{2}\rangle -{\langle M\rangle }^{2}\right)\;,$$

where 〈M〉 may be included in the self-consistent effective field but is considered to be *fixed*, i.e., contributions from the self-consistent field are *not* included in Equation (9).

The magnetic susceptibility should be defined by

(10) $$\chi_{\mathrm{MF}}=\frac{\partial }{\partial h}\;\langle M\rangle$$

within the interchain mean-field approximation. Insertion of the definition Equation (8) for the magnetization and some straightforward algebra leads to

(11) $$\chi_{\mathrm{MF}}=\left(1-J_{⊥}\;\chi_{\mathrm{MF}}\right)\;\chi_{1D}\;.$$

The result (11) can be solved for $\chi_{\mathrm{MF}}$ and one finds (As a consequence of the spin inversion that we have applied to half of the chains at h=0, the following is actually not the uniform, but a staggered susceptibility in the case of a vanishing external field.)

(12) $$\chi_{\mathrm{MF}}=\frac{\chi_{1D}}{1+J_{⊥}\;\chi_{1D}}\;.$$

This approximation is widely used in the literature (see for example [57,58,59]) and also known under the name “random phase approximation”. Since there are some similarities with the Stoner model of ferromagnetism (see, e.g., chapter 7.4 of [60]), one can also call $1+J_{⊥}\;\chi_{1D}$ a “Stoner factor”. Note that the above derivation is essentially the same as the computation on page 66 of [61], but the linearizing assumption $\langle M\rangle \approx h\;\chi_{\mathrm{MF}}$ has been dropped. Accordingly, we see that Equation (12) also applies for a finite magnetization 〈M〉≠0 of a single chain.

A zero of the denominator in Equation (12) signals a second-order phase transition. This yields the standard condition for the Néel temperature

(13) $$-J_{⊥}=\frac{1}{\chi_{1D}\left(T_{N}\right)}\;.$$

Let us use this condition to take a look at the ordering transition in zero external field where 〈M〉=0 for $\;T>T_{N}$ such that Equation (13) can be evaluated without running a self-consistency cycle. Our QMC results for $1/\chi_{1D}$ at h=0 are shown in Figure 9 for N=100, 200, and 400. The fact that these three system sizes are essentially indistinguishable on the scale of the figure shows that N=100 suffices to represent the thermodynamic limit N=∞.

If one assumes the value $-J_{⊥}=0.072$ K (horizontal line in Figure 9) that has been deduced in [28] by fitting the magnetic susceptibility for T≥10 K, one reads off an ordering temperature $T_{N}\approx 0.77\;J\approx 2.16$ K. This deviates by about 12% from the experimental value $T_{N}=2.45$ K, which is remarkably good for a mean-field theory. Conversely, if one insists on the experimental value $T_{N}=0.875\;J$, one infers an interchain coupling $-J_{⊥}\approx 0.04\;J\equiv 0.11$ K, which is about 50% larger than the estimate of [28]. In fact, $1/\chi_{1D}$ varies quite strongly in this temperature range. Therefore, $T_{N}$ is not very sensitive to the interchain coupling $J_{⊥}$.

In any case, an interchain coupling of a few percent suffices to yield an antiferromagnetic ordering temperature at h=0 that is of the same order as the coupling in an individual chain, reflecting strong ferromagnetic ordering tendencies of the decoupled chains.

#### 3.3.2. Specific Heat

Let us now take a closer look at the specific heat in interchain mean-field theory. As in the case of the magnetic susceptibility, the numerical treatment of the individual chains provides convenient access to

(14) $$c_{1D}=\frac{\beta^{2}}{N}\;\left(\langle {\left(H^{\mathrm{MF}}\right)}^{2}\rangle -{\langle H^{\mathrm{MF}}\rangle }^{2}\right)\;,$$

where 〈M〉 may again be included in the self-consistent effective field but is considered to be *fixed*.

The self-consistent magnetization 〈M〉 is also temperature-dependent such that the specific heat should be written as a first derivative of the internal energy

(15) $$c_{\mathrm{MF}}=\frac{1}{N}\;\frac{\partial \;U}{\partial T}\;=\frac{1}{N}\;\frac{\partial }{\partial T}\;\left(\langle H^{\mathrm{MF}}\rangle -N\;\frac{J_{⊥}}{2}{\langle M\rangle }^{2}\right)\;.$$

The temperature derivative can in principle be calculated numerically. For reasons of numerical stability, particularly in a Monte-Carlo setting, it is nevertheless preferable to carry the derivatives out analytically. Since we are not aware of such an analysis having been presented before, we present it here in some detail. With the help of $[M,H^{\mathrm{MF}}]=0$, we find from Equation (15) that

(16) $$\begin{array}{ccc} c_{\mathrm{MF}} & = & -\frac{\beta^{2}}{N}\;\frac{\partial }{\partial \beta }\;\left(\langle H^{\mathrm{MF}}\rangle -N\;\frac{J_{⊥}}{2}{\langle M\rangle }^{2}\right)\\ & = & c_{1D}+\beta^{3}\;J_{⊥}\;\frac{\partial \;\langle M\rangle }{\partial \beta }\;\left(\langle H^{\mathrm{MF}}\;M\rangle -\langle H^{\mathrm{MF}}\rangle \langle M\rangle \right)\;. \end{array}$$

This expression contains another derivative $\frac{\partial \;\langle M\rangle }{\partial \beta }$ for which we can find an expression that is very similar to Equation (12) (including a “Stoner factor” $1+J_{⊥}\;\chi_{1D}$):

(17) $$\frac{\partial \;\langle M\rangle }{\partial \beta }=-\frac{\langle H^{\mathrm{MF}}\;M\rangle -\langle H^{\mathrm{MF}}\rangle \langle M\rangle }{1+J_{⊥}\;\chi_{1D}}\;.$$

Noting the relation

(18) $${\left.\frac{\partial \;\langle M\rangle }{\partial T}\right|}_{h,1D}=\beta^{2}\;\left(\langle H^{\mathrm{MF}}\;M\rangle -\langle H^{\mathrm{MF}}\rangle \langle M\rangle \right)\;,$$

the combination of Equations (16) and (17) can also be written in the following form:

(19) $$c_{\mathrm{MF}}=c_{1D}-\frac{J_{⊥}}{\beta }\frac{1}{1+J_{⊥}\;\chi_{1D}}\;{\left({\left.\frac{\partial \;\langle M\rangle }{\partial T}\right|}_{h,1D}\right)}^{2}$$

In this form, the sign of the second term is evident. This form is also useful for the purpose of evaluation since Equation (19) contains only quantities that can be related to static expectation values for a single chain with a *fixed* value of 〈M〉 via Equations (9), (14), and (18). The only object that is non-standard is the crosscorrelator in Equation (18); however, it represents exactly the same observable as was used in Ref. [62] to compute the adiabatic cooling rate by QMC.

#### 3.3.3. Comparison with Experimental Specific Heat

**Figure 10:**
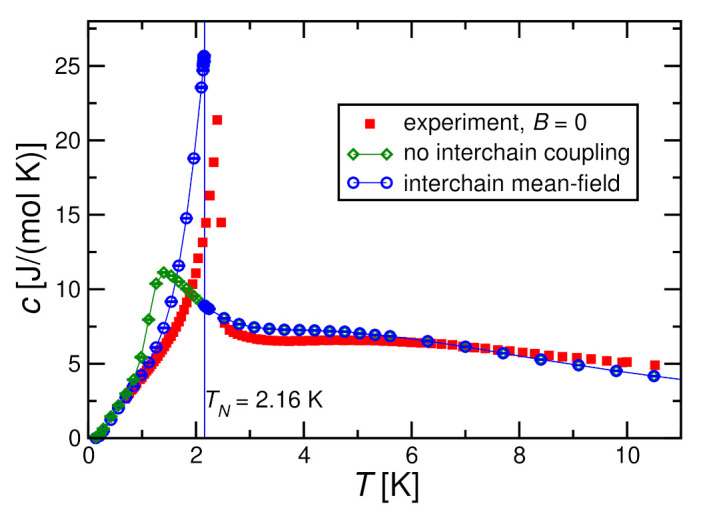
Magnetic specific heat per spin *c* for J=2.8 K, D=0.36 K in zero magnetic field h=0 in comparison with the experimental results for B=0. Theoretical results are obtained by QMC with N=100 both for decoupled chains ($J_{⊥}=0$) and with a self-consistent mean-field treatment for $J_{⊥}=-0.072$ K.

**Figure 11:**
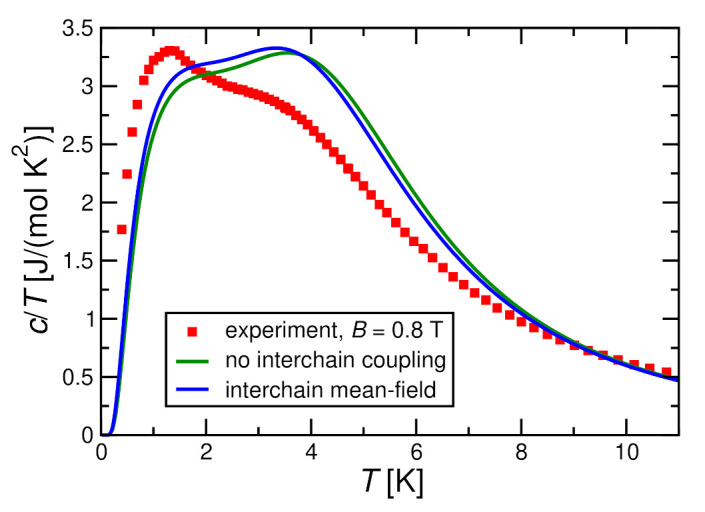
Magnetic specific heat per spin divided by temperature c/T for J=2.8 K, D=0.36 K in a magnetic field h=0.4J in comparison with the experimental results for B=0.8 T. Theoretical results are obtained by ED with N=10 both for decoupled chains ($J_{⊥}=0$) and with a self-consistent mean-field treatment for $J_{⊥}=0.072$ K.

**Figure 12:**
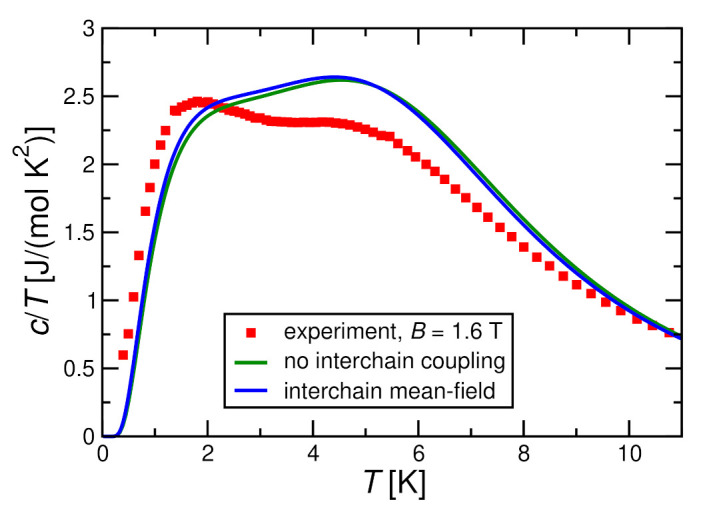
Magnetic specific heat per spin divided by temperature c/T for J=2.8 K, D=0.36 K in a magnetic field h=0.8J in comparison with the experimental results for B=1.6 T. As in Figure 11, theoretical results are obtained by ED with N=10 both for decoupled chains ($J_{⊥}=0$) and with a self-consistent mean-field treatment for $J_{⊥}=0.072$ K.

We are now in a position to perform a comparison with the experimental results for the specific heat of Figure 3. Figure 10, Figure 11 and Figure 12 show the results for h=0, 0.4J, and 0.8J (corresponding to the experimental magnetic fields B=0, 0.8 T, and 1.6 T, respectively). For h=0, we used QMC with N=100, while for h=0.4J and 0.8J, we used ED with N=10. These systems sizes should be sufficiently large to render finite-size effects negligible according to the discussions in Section 3.2. From a technical point of view, we note that at h=0 and in the paramagnetic phase, 〈M〉=0 such that $\frac{\partial \;\langle M\rangle }{\partial T}=0$ and the correction term in Equation (19) vanishes, i.e., $c_{\mathrm{MF}}=c_{1D}$, and the blue circles are identical to the green diamonds in Figure 10 for $T>T_{N}$.

Figure 11 and Figure 12 show that the interchain coupling leads only to small corrections for a magnetic field h≥0.4J; the trend is towards the experimental data, but the shift by interchain coupling does not change the situation significantly. Nevertheless, the two theory curves and the experimental one in Figure 11 and Figure 12 exhibit double-peak structures where the two peaks are located at very similar temperatures between theory and experiment.

Figure 10 demonstrates that in zero field (h=0), interchain coupling is not only essential for reproducing the ordering transition to good accuracy, as we have seen before, but that thanks to the “Stoner factor”, the correction term in (19) dominates the specific heat just below the ordering transition and thus gives rise to the characteristic ordering peak. We note, however, that the singularity in the denominator of Equation (19) is deceptive since the numerator (18) also vanishes such that *c* has a finite limit for $T↗T_{N}$. Consequently, our interchain mean-field theory remains in the universality class of Landau theory [63] with a specific heat exponent α=0.

## 4. Magnetocaloric Properties

The strong dependence of the specific heat of MnNi(NO${}_{2}$)${}_{4}$(en)${}_{2}$ on an applied magnetic field promises a strong magnetocaloric effect and potential relevance to low-temperature magnetic refrigeration by adiabatic demagnetization, e.g., see Refs. [64,65]. Therefore, let us have a closer look at its magnetocaloric properties.

**Figure 13:**
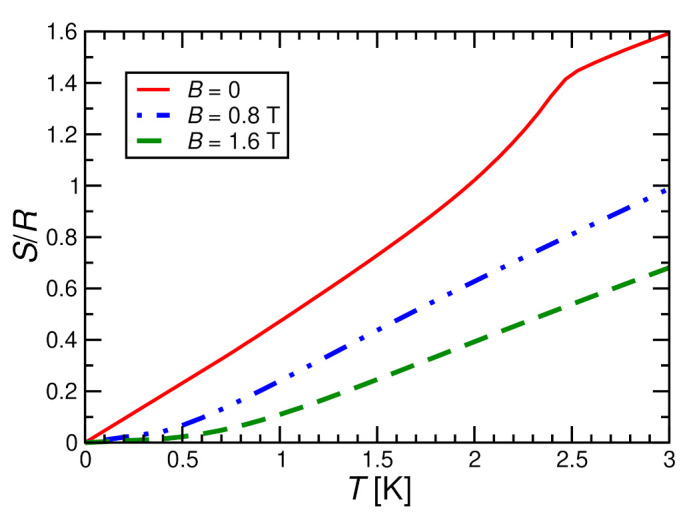
Magnetic entropy per mole of MnNi(NO${}_{2}$)${}_{4}$(en)${}_{2}$ in magnetic fields of B=0, 0.8, and 1.6 T, respectively.

Figure 13 shows the experimental magnetic entropy that is obtained by integrating the experimental results for the specific heat $c_{p,mag}/T$ of Figure 3b with respect to temperature *T*. The B=0 curve corresponds to the one shown already in Figure 2b. Figure 13 shows that the magnetic entropy is significantly reduced by applying a magnetic field of B=1.6 T, or even 0.8 T, corresponding to polarization of the spin system by the applied magnetic field. Consequently, we expect cooling of the spin system during adiabatic demagnetization. Let us consider, for example, an ideal adiabatic process that starts with T=1.5 K for B=1.6 T. We read off from Figure 13 that the same entropy is found at B=0 for T≈0.5 K, i.e., adiabatic demagnetization from B=1.6 T to B=0 would cool from an initial temperature T=1.5 K to a final temperature of T≈0.5 K. Likewise, an ideal adiabatic process starting with T=2.5 K at B=1.6 T would cool to T≈1.1 K during a single ideal adiabatic demagnetization process. These are relatively large effects in the liquid Helium range, which is also remarkable since one is cooling through a phase transition into a magnetically ordered state. The main caveat is that the processes of the two examples exploit only 8% or 19% of the total magnetic entropy S≈2.89R in the first and second case, respectively.

**Figure 14:**
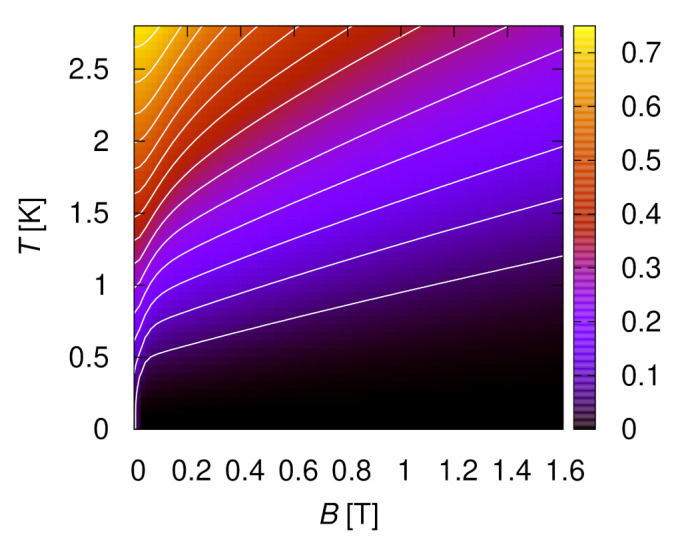
Entropy *S* per spin calculated by ED for an isolated chain with N=10 spins and J=2.8 K, D=0.36 K.

Next, let us comment on a numerical description. The entropy is not directly accessible in QMC simulations such that we resort to ED even if this leads to stronger finite-size effects. Furthermore, for the full *h* and *T* dependence of the magnetic entropy *S*, we would have to model the ordered state in an external magnetic field (gray shaded region in the inset of Figure 3b). However, this is expected to correspond to a canted spin configuration and is thus beyond the present investigation. Therefore, we also neglect interchain coupling, i.e., we focus on a situation corresponding to the one discussed in Section 3.2 (see, however, Appendix C for a discussion of simple single-site mean-field theory). Figure 14 shows the corresponding result for the entropy (now normalized per spin) of an N=10 chain. This density plot of S(B,T) permits to immediately read off the magnetocaloric effect. In particular, the isentropes, corresponding to the white lines in Figure 14, directly show the behavior under an adiabatic process. Finite-size effects are expected to be small for B≥0.8 T (corresponding to h/J≥0.4, compare Figure 6 and Figure 7), but they are known to be relevant throughout the temperature range of Figure 14 for B=0 (h=0, compare Figure 5).

**Figure A4:**
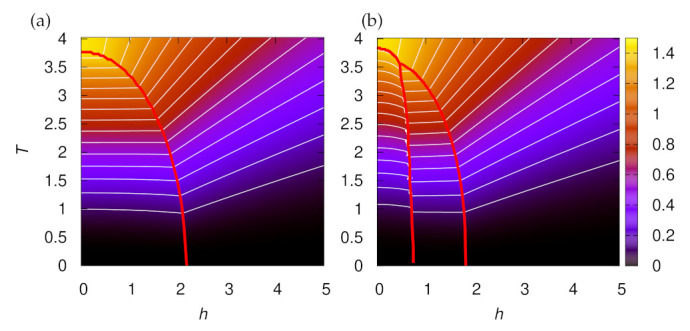
Single-site mean-field results for the entropy per spin at J=1, $J_{⊥}=0.5$, and D=0 (**a**) and at D=0.1 (**b**). Red lines are the phase boundaries from Figure A3.

We also read off cooling by adiabatic magnetization from Figure 14 with a size of the effect corresponding to the experimental data of Figure 13. Note that the entropy is normalized to mole in Figure 13 and per spin in Figure 14, amounting to a factor two difference in addition to the factor *R*. Since we have ferromagnetic chains, the strongest cooling occurs in Figure 14 upon approaching a zero external field. If one adds antiferromagnetic interchain coupling, we expect to recover the magnetically ordered phase that is observed experimentally for B<0.4 T (compare inset of Figure 3b) and then cooling might actually occur when entering this phase (compare Figure A4 and the related discussion in Appendix C for the behavior in single-site mean-field theory). Indeed, Figure 10 demonstrates that interchain coupling reshuffles entropy from low temperatures to the ordering transition such that the most significant cooling probably occurs around it.

## 5. Conclusions and Perspectives

We carried out specific heat measurement in zero and applied fields on the bimetallic chain compound MnNi(NO${}_{2}$)${}_{4}$(en)${}_{2}$. By determining the lattice contribution of the specific heat, we have extracted the magnetic specific heat $c_{p,mag}$. For the first time, in its temperature dependence, we verify a long-predicted double-peak-like structure. Comparison with numerical calculations for the bimetallic $S_{1}=1$, $S_{2}=5/2$ ferromagnetic spin chain yields a very close resemblance on a semi-quantitative level.

Alternating spins are not the only mechanism that may give rise to a double-peak structure in the specific heat. For example, also a ferromagnetic S=1 chain alone can give rise to such structures when subjected to a strong single-ion anisotropy *D* [66]. However, the numerical data of Section 3.2 (and further results that we do not show here) demonstrate that these two features are already present at D=0 in the $S_{1}=1$, $S_{2}=5/2$ chain and can be traced to finite *D* even if the presence of a single-ion anisotropy does affect the behavior of the specific heat at a quantitative level. Hence, we conclude that our experimental observation of a double-peak-like structure in the specific heat directly reflects the alternating spins $S_{1}=1$ and $S_{2}=5/2$ along the chains. The application of an external magnetic field to MnNi(NO${}_{2}$)${}_{4}$(en)${}_{2}$ is essential to suppress magnetic order and, thus, reveal this double-peak feature experimentally.

The ordered phase that is observed in MnNi(NO${}_{2}$)${}_{4}$(en)${}_{2}$ for low temperatures and small applied magnetic fields is due to an antiferromagnetic interchain coupling. Although its absolute value is much smaller than the ferromagnetic coupling along the chains, it has a strong effect at low temperatures and in the absence of a magnetic field. In order to describe this ordered phase, we developed a mean-field treatment of interchain coupling. The combination of QMC simulations for isolated chains and such an interchain mean-field theory not only yields a remarkably accurate value for the ordering transition temperature $T_{N}$ using previously determined parameters [28] but also yields excellent agreement for the full temperature dependence of the magnetic specific heat. For fields h≥0.4J, the mean-field corrections are small, reflecting the smallness of the interchain coupling constant $J_{⊥}$.

Beyond the very close resemblance on a qualitative level, there are some quantitative differences between experiment and theory. For instance, while in the calculations the maximum of $c_{p,mag}/T$ is found close to $T_{\mathrm{up}}$, in the experiments it is observed at $T_{\mathrm{low}}$. These small differences may be due to the single-ion anisotropy being located on both the Ni and Mn sites, and not just the Mn ones, or effects of interchain coupling beyond mean-field theory. However, a further refinement of the model would require additional information about the excitation spectrum such as inelastic neutron scattering.

Another theoretical challenge concerns the theoretical description of the ordered state in a magnetic field. For h=0 and strong fields along the anisotropy axis (h≥0.4), one may restrict the discussion to magnetization along the *z*-axis only. However, for a magnetic field applied at an angle to the anisotropy axis, and also for ordered phases where the ordered moment cants away from the field/anisotropy axis, it will in general be necessary to replace the last term in (4) by vectors, i.e., by $\left(\vec{h}-\sum_{j\neq i}J_{i,j}\;\langle {\vec{M}}_{j}\rangle \right)\cdot \sum_{x=1}^{N/2}\left({\vec{S}}_{x}+{\vec{s}}_{x}\right)$. This generalization can be implemented in single-site mean-field theory; however, such a strong approximation fails to be quantitatively accurate for the present situation (compare Appendix C). By contrast, such generic field directions in the interchain mean-field theory of Section 3.3 break conservation of total $S^{z}$, render the computations even more challenging, and thus go beyond the present investigation.

Finally, we have shown that the strong sensitivity of MnNi(NO${}_{2}$)${}_{4}$(en)${}_{2}$ to even small applied magnetic fields gives rise to a strong magnetocaloric effect, i.e., large cooling by adiabatic demagnetization from initial fields *B* on the order of 1 T. Even if the magnetic entropy of MnNi(NO${}_{2}$)${}_{4}$(en)${}_{2}$ may be a bit small for practical applications in the temperature range of interest, this observation suggests materials with competing strong ferromagnetic and weaker antiferromagnetic interactions as promising candidates for efficient low-temperature refrigeration.

## Figures and Tables

**Figure A1:**
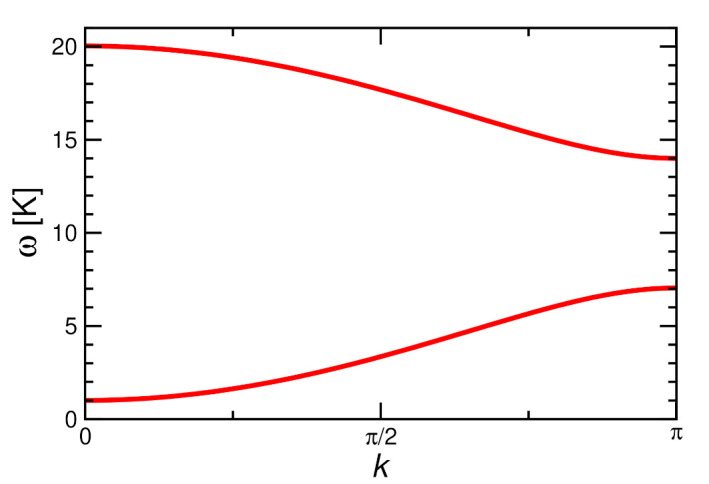
The one-magnon dispersion (A2) and (A3) for J=2.8 K, D=0.36 K, and d=0.

**Figure A2:**
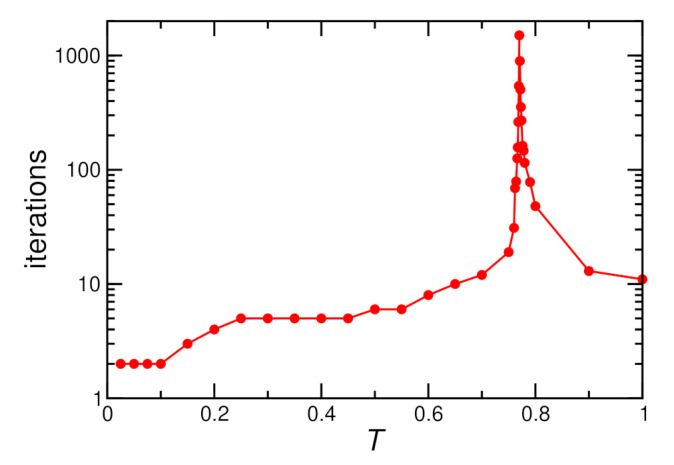
Number of iterations performed for the N=100 QMC data in Figure 8 (recall that J=1). Actual data are indicated by filled circles; lines are guides to the eye.

**Figure A3:**
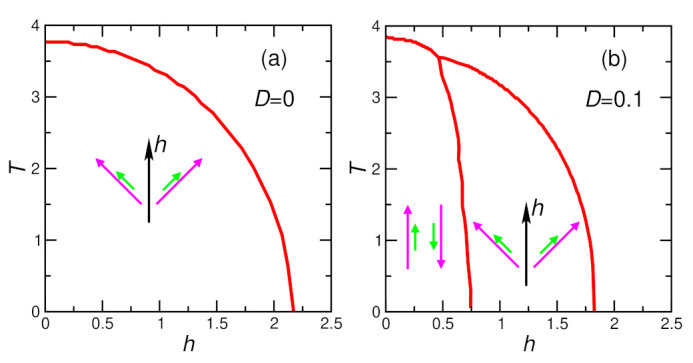
Single-site mean-field phase diagrams for J=1, $J_{⊥}=0.5$, and D=0 (**a**) and for D=0.1 (**b**). The schematics indicate the spin configurations relative to the applied magnetic field in the ordered phases. A pair of parallel arrows indicates Mn (magenta) and Ni (green) spins in one chain; the second pair indicates the neighboring chain. The phase at high temperatures or large magnetic fields is paramagnetic.

## Data Availability

The data contained in this publication is available from the corresponding author upon reasonable request.

## Appendix A. One-Magnon Dispersion

Let us generalize the computation of the one-magnon dispersion of Ref. [28] to the presence of single-ion anisotropies. To this end, we generalize the chain model (1) to include anisotropy terms on both Mn and Ni sites but drop the magnetic field term:

(A1) $$H=-J\sum_{x=1}^{N/2}\left({\vec{S}}_{x}\cdot {\vec{s}}_{x}+{\vec{s}}_{x}\cdot {\vec{S}}_{x+1}\right)-D\sum_{x=1}^{N/2}{\left(S_{x}^{z}\right)}^{2}-d\sum_{x=1}^{N/2}{\left(s_{x}^{z}\right)}^{2}\;.$$

Since the coupling along the chain is ferromagnetic, the ground state is also ferromagnetic. A *D*, d>0 selects the two maximally polarized components of the ground state. The one-magnon sector is then obtained by flipping a single spin relative to this polarized state. This is a single-particle problem that is straightforward to solve by Fourier transformation and diagonalization of the 2×2 matrix resulting from the two-site unit cell. This yields two branches of one-magnon excitation energies

(A2) $$\begin{array}{ccc} \omega_{\pm }\left(k\right) & \;\;=\;\; & J\;\left(S_{2}+S_{1}\right)+A\left(S_{2}\right)+B\left(S_{1}\right)\\ & & \pm \sqrt{J^{2}\;\left(S_{2}^{2}+S_{1}^{2}+2\;S_{2}\;S_{1}\;\cos\left(k\right)\right)+2\;J\;\Delta \left(S_{1},S_{2}\right)\;\left(S_{1}-S_{2}\right)+\Delta {\left(S_{1},S_{2}\right)}^{2}} \end{array}$$

with

(A3) $$A\left(S_{2}\right)=\frac{2\;S_{2}-1}{2}\;D\;,\;B\left(S_{1}\right)=\frac{2\;S_{1}-1}{2}\;d\;,\;\Delta \left(S_{1},S_{2}\right)=A\left(S_{2}\right)-B\left(S_{1}\right)\;.$$

Figure A1 shows the two branches of the one-magnon dispersion $\omega_{\pm }\left(k\right)$ for the model (1) and the parameters that we have used in the main text. The most important qualitative difference to the previous analysis in Ref. [28] is the opening of a gap $\omega_{-}\left(0\right)\;\approx \;1$ K due to the single-ion anisotropy D=0.36 K. Further inspection of (A2), (A3) shows that a reshuffling of the Mn anisotropy to the Ni one has a significant effect on the gap $\omega_{-}\left(0\right)$. For example, the parameters J=2.8 K, D=0, and d=1.44 K would conserve $A\left(S_{2}\right)+B\left(S_{1}\right)$ and yield an overall picture that is very similar to Figure A1 but a reduced gap $\omega_{-}\left(0\right)\approx 0.4$ K. Such a reduction of the gap may indeed be consistent with the experimental data in Figure 11 and Figure 12, but one would need an accurate experimental estimate of the gap for a more precise statement.

## Appendix B. Details of Self-Consistency Procedure in QMC

Figure A2 shows the number of iterations performed in order to reach self-consistency for the N=100 QMC data presented in Figure 8. The precise number of iterations depends on details such as the desired level of accuracy (we aimed at reducing the error on 〈M〉 to below $10^{-3}$) and the exact way the iterations are run. One can nevertheless draw some qualitative conclusions: sufficiently far away from the Néel temperature, self-consistency is obtained after a few iterations but the number of required iterations explodes upon approaching the phase transition, a phenomenon that may be interpreted as a form of “critical slowing down”. Bearing in mind that it may take a few days to obtain sufficiently small statistical error bars within a single iteration and that several hundred to more than 1000 iterations have been performed, it is also evident that the computations have been running over an extended period of time. The procedure could be sped up by a more sophisticated root-finding algorithm than simple iteration, but we stayed with the latter for the present investigation.

## Appendix C. Single-Site Mean-Field Approximation

In order to theoretically explore the ordered phase in small external magnetic fields where the spins are expected to be canted, we summarize here a complete single-site mean-field decoupling of the Hamiltonian (1) supplemented by the interchain coupling (2); for further details, we refer to chapter 5 of Ref. [67].

Since the days of Pierre Weiss [68], the mean-field approximation has become a textbook method in the theory of magnetism (see, e.g., Refs. [69,70] and references therein) such that we will comment only briefly on it. The essential step is to replace the terms in Equation (1) as follows:

(A4) $${\vec{S}}_{x}\cdot {\vec{s}}_{y}\;\to \;{\vec{S}}_{x}\cdot \langle {\vec{s}}_{y}\rangle +\langle {\vec{S}}_{x}\rangle \cdot {\vec{s}}_{y}-\langle {\vec{S}}_{x}\rangle \cdot \langle {\vec{s}}_{y}\rangle \;.$$

In combination with the mean-field decoupling of the interchain coupling (4), this leads to a set of single-spin problems with individual coupling to the external magnetic field, possibly single-ion anisotropy, and coupling to their neighbors taken into account effectively via an additional mean field. However, the expectation values $\langle {\vec{S}}_{x}\rangle$ and $\langle {\vec{s}}_{y}\rangle$ need to be determined self-consistently for this set of coupled problems. We solve this self-consistency condition by iteration, i.e., we assume a configuration of the $\langle {\vec{S}}_{x}\rangle$ and $\langle {\vec{s}}_{y}\rangle$, solve the single-ion problems numerically, recompute the expectation values, and iterate until convergence. In principle, the procedure can be implemented for a lattice of coupled mean-field problems (see, e.g., Refs. [56,71]). However, we make some further plausible assumption in order to reduce the numerical effort. Firstly, in view of the ferromagnetic coupling along the chain, we assume the pattern to be translationally invariant, although we do need two mean fields due to the alternating spins. Secondly, in view of the antiferromagnetic interchain coupling, we allow for two inequivalent chains. This leads to a set of four mean-field coupled single-ion problems. Finally, we assume the spin configuration to lie in a plane that includes the external magnetic field (and thus, also the single-ion anisotropy that we assume to be parallel to the magnetic field).

It turns out that there is no finite-field phase in the parameter regime studied in the main text. This may be attributed to the antiferromagnetic interchain coupling $J_{⊥}$ just partially canceling the ferromagnetic chain coupling *J* when all couplings are treated at the mean-field level, thus leading to an effectively ferromagnetic system with just a reduced effective coupling constant. Therefore, we use modified parameters J=1, $J_{⊥}=0.5$, D=0.1 in this appendix and focus on the qualitative behavior.

### Appendix C.1. Phase Diagram

Figure A3 shows the mean-field phase diagram with and without the single-ion anisotropy. The phase diagrams were obtained from an analysis of the spin configurations [67]. In the case of D=0 (Figure A3a), we find an ordered antiferromagnetic phase. At zero field h=0, the direction of the ordering vector is arbitrary. Application of a small field orients the spins orthogonal to the field direction. Upon increasing the magnetic field, spins are increasingly tilted towards the field direction. The overall behavior is very similar to the well-known Heisenberg antiferromagnet on a bipartite lattice (see, e.g., Ref. [72] for the case of the square lattice), the main difference being that all Mn and Ni spins in one chain adopt the role of those of one sublattice. Note that in the case of D=0, the Mermin–Wagner theorem [73] would forbid a finite-temperate transition in the case of one or even two dimensions. The phase diagram of Figure A3a should thus be thought of to represent the case of chains coupled in three dimensions.

Figure A3b presents the phase diagram for a single-ion anisotropy D>0. The main difference with the D=0 case is the appearance of an additional phase at small magnetic fields. Indeed, the single-ion anisotropy pins the spins along the anisotropy axis. For a small magnetic field (that we choose here to be parallel to the anisotropy axis), the spins remain pinned along this axis and a finite critical field is needed to enter the “spin-flop” phase where the spins cant towards the magnetic field and that we already observed for the case D=0 (Figure A3a). The structure of the phase diagram in Figure A3b is again reminiscent of the well-known phase diagram of an anisotropic antiferromagnet on a bipartite lattice (see, e.g., Refs. [69,74,75,76,77]). The main difference is again that the Ni ($S_{1}=1$) and Mn ($S_{2}=5/2$) spins of one chain pair up to correspond to one sublattice. Actually, when the spins tilt with respect to the magnetic field, Ni and Mn ones are not expected to be exactly parallel to each other, particularly if the Mn one is subject to a single-ion anisotropy while the Ni one is not. However, it turns out that the angle between a pair does not exceed a few degrees [67]. Accordingly, the sketches of the spin configurations in Figure A3 are schematic in the sense that spin pairs are almost but not necessarily exactly parallel. For D>0, the Mermin–Wagner theorem [73] allows finite-temperature ordering starting in two dimensions. Nevertheless, for small values of *D* and weakly coupled ferromagnetic chains, i.e., the situation relevant to MnNi(NO${}_{2}$)${}_{4}$(en)${}_{2}$, one is still close to a situation where ordering would be forbidden such that the present mean-field theory is likely to overestimate the transition temperature. Indeed, at h=0, the estimate inferred from Figure 9 is $T_{N}<J$ rather than $T_{N}\approx 4\;J$, as observed in Figure A3.

Let us briefly comment on a comparison to the experimental phase diagram. The inset of Figure 3 just shows the transition into an ordered phase. However, the experimental data for the magnetization and Bragg intensity of elastic neutron scattering show two features at T=1.8 K as a function of applied field *B* [47]. We believe that these two experimental features correspond to the two transitions in the mean-field phase diagram Figure A3b.

### Appendix C.2. Entropy and Magnetocaloric Effect

Now, we turn to the magnetocaloric properties. Mean-field theory has been used before for this purpose (see, e.g., Refs. [78,79,80,81]). Indeed, once the self-consistent mean-field solution is known, both the free energy *F* and the internal energy *U* are straightforward to compute; from these, one obtains the entropy via

(A5) $$S=\frac{U-F}{T}\;.$$

Figure A4 presents results of the entropy for our mixed-spin system with D=0 (a) and D=0.1 (b). The representation is analogous to Figure 14 with entropy being normalized per spin and white lines denoting isentropes. Furthermore, we superimpose the phase transitions from Figure A3 as red lines in Figure A4. One observes that the phase boundaries correspond to kinks in the isentropes. In fact, the isentropes of Figure A4b are discontinuous across the transition separating the collinear antiferromagnetic phase at low magnetic fields and the spin-flop phase at higher fields, reflecting the first-order nature of this transition.

The main qualitative finding is that we observe cooling by adiabatic demagnetization, as expected. However, the effect is mainly restricted to the paramagnetic phase. Upon entering the ordered (spin-flop) phase, the isentropes become very flat, i.e., temperature *T* varies very little when *h* is varied in this ordered phase. There is even a small heating effect when *h* is lowered through the transition between the spin-flop and collinear ordered phase that appears for D=0.1 (Figure A4b). A quantitative comparison with MnNi(NO${}_{2}$)${}_{4}$(en)${}_{2}$ is unfortunately precluded, e.g., by mean-field theory overestimating ordering tendencies, as discussed before in the context of Figure A3. Let us note a final peculiarity of mean-field theory, namely, that the total entropy of the system is essentially recovered for $T>T_{N}$ at h=0 and thus visible in Figure A4: Indeed, symmetry arguments imply that the mean-field correction to the specific heat *c* vanishes at h=0 for $T>T_{N}$, as already stated in Section 3.3.3. As a consequence, the total entropy is recovered exactly for D=0. For D≠0, the single-ion splitting leads to some fluctuations surviving for $T>T_{N}$. However, for D=0.1J, the effect is so small that less than 1 per mille of the total entropy is missing at the highest temperature (shown in Figure A4b) while it would be recovered only in the limit T→∞ for the case of the ED result of Figure 14. Furthermore, we recall that the experiment recovers barely half of the total entropy at $T_{N}$ for B=0 (compare Figure 2b and the related discussion in Section 2.2).

Let us conclude this discussion by mentioning that the magnetic susceptibility χ and specific heat *c* can, in principle, also be investigated within the single-site mean-field approximation. However, if one wants to avoid numerical derivatives, the presence of two inequivalent sites in each chain requires matrix generalizations of Equations (12) and (19). We refer to Ref. [67] for further details on these aspects.
